# Ultrasound-Guided Low-Dose Hyaluronidase for Infraorbital Artery Occlusion with Secondary Gingival Ischemia After Hyaluronic Acid Filler Injection: A Case Report

**DOI:** 10.3390/diagnostics16131973

**Published:** 2026-06-25

**Authors:** Carla Barber-García, Endika Nevado-Sánchez, Sandra Núñez-Rodríguez, Alejo Cavadas, Andrea Bueno-de la Fuente, Jerónimo Javier González-Bernal

**Affiliations:** 1 Clínicas Carla Barber, 28002 Madrid, Spain; drcarlabarber@clinicasbarber.com; 2 Endika Nevado Cirugía Pl’stica y Estética, 09004 Burgos, Spain; endineva@hotmail.com; 3 Department of Health Sciences, Universidad Isabel I, 09001 Burgos, Spain; sandra.nunez@ui1.es; 4 Santa Clara Health Center, 09002 Burgos, Spain; andreabuenodf@gmail.com; 5 Department of Health Sciences, University of Burgos, 09001 Burgos, Spain; jejavier@ubu.es

**Keywords:** hyaluronic acid filler complications, vascular occlusion, infraorbital artery, anterior superior alveolar artery, high-frequency ultrasound, image-guided intervention, hyaluronidase

## Abstract

**Background and Clinical Significance**: Hyaluronic acid fillers are currently the most widely used materials in aesthetic medicine and represent one of the most frequently performed minimally invasive procedures worldwide. Vascular occlusion is the most severe complication associated with this type if filler injections due to the risk of tissue necrosis and permanent sequelae. Early recognition and precise identification of the affected vascular territory are essential to prevent irreversible damage. **Case Presentation**: his report describes a case of infraorbital artery occlusion with retrograde extension to the anterior superior alveolar artery and associated gingival ischemia, highlighting the role of high-frequency ultrasound in diagnosis and management. A 60-year-old woman developed vascular occlusion following supraperiosteal HA injection in the medial cheek. Clinical findings included livedo reticularis in the infraorbital and nasal regions, along with ipsilateral gingival anesthesia and mucosal ischemia. High-frequency ultrasound was used to assess the extent and mechanism of vascular involvement. A targeted treatment approach was implemented using low-dose hyaluronidase (100 IU/mL), with 200 IU administered in the infraorbital region and an additional 100 IU delivered under ultrasound guidance to the affected alveolar branch. Ultrasound examination revealed extrinsic compression of the infraorbital artery and secondary occlusion of the anterior superior alveolar artery consistent with retrograde embolization. Following image-guided administration of hyaluronidase, complete reperfusion was achieved, with resolution of both cutaneous and gingival ischemia and no functional or aesthetic sequelae. **Conclusions**: High-frequency ultrasound provides critical diagnostic information in vascular complications after HA filler injection, allowing for accurate identification of the mechanism and extent of vascular involvement. Ultrasound-guided low-dose hyaluronidase may represent an effective and safe strategy to restore perfusion while minimizing unnecessary enzyme exposure and associated adverse effects.

## 1. Introduction

Hyaluronic acid (HA) fillers are currently the most widely used materials in aesthetic medicine and represent one of the most frequently performed minimally invasive procedures worldwide [1,2]. Their favorable safety profile and reversibility have contributed to their widespread use. However, the increasing number of procedures has been accompanied by a proportional rise in adverse events [2,3]. While most complications are mild and self-limiting, vascular occlusion remains the most severe due to its potential to cause tissue necrosis, visual impairment, and permanent functional or aesthetic sequelae [4,5].

The pathophysiology of HA-related vascular occlusion involves either inadvertent intravascular injection with subsequent embolization or extrinsic compression of vascular structures by periarterial filler deposits [6,7]. These mechanisms may lead to impaired perfusion within the affected angiosome and, in some cases, to distal ischemic involvement through anterograde or retrograde embolization [7]. The clinical presentation typically includes early warning signs such as disproportionate pain, pallor, livedo reticularis, and delayed capillary refill [6,8]. However, the variability in vascular anatomy and the complexity of facial arterial networks, particularly in the midface region, can make it difficult to accurately identify the affected vessel and the true extent of ischemic involvement based solely on clinical findings [9].

In this context, high-frequency ultrasound has emerged as a valuable tool in aesthetic and dermatologic practice, enabling real-time visualization of soft tissue structures, vascular anatomy, and filler deposits [10,11]. Ultrasound can assist in differentiating between intravascular obstruction and extrinsic compression, as well as in detecting secondary embolization patterns that may not be clinically evident [10]. Moreover, its use allows for image-guided therapeutic interventions, facilitating precise delivery of hyaluronidase to the affected areas and potentially reducing the need for high cumulative doses [11].

Despite these advantages, detailed descriptions of ultrasound findings in cases of infraorbital artery occlusion with secondary involvement of the anterior superior alveolar artery remain scarce. In particular, gingival ischemia as a manifestation of retrograde embolization has been rarely reported, and its recognition may be challenging without imaging support [12].

Therefore, the aim of this report is to describe a case of infraorbital artery occlusion following HA filler injection, with retrograde extension to the anterior superior alveolar artery and associated gingival ischemia, highlighting the diagnostic value of high-frequency ultrasound and its role in guiding a targeted low-dose hyaluronidase treatment strategy.

## 2. Case Report

A 60-year-old woman underwent hyaluronic acid (HA) filler treatment in September 2025 to restore midface volume and improve structural support. At a scheduled follow-up two months later, a residual volume deficit in the medial malar region was identified, and a secondary injection was performed.

The procedure was performed as follows: Following standard skin antisepsis with 2% alcoholic chlorhexidine, 0.2 mL of cross-linked HA was injected into the right medial cheek and 0.1 mL into the left medial cheek (Fillmed Universal, Laboratoires Fillmed, France). The filler was administered using a 30 G, 13 mm needle in a supraperiosteal plane. No aspiration was performed. The procedure was uneventful, with no immediate signs of vascular compromise, including absence of pain, livedo reticularis, or alterations in capillary refill.

Approximately six hours after the procedure, the patient reported progressive skin discoloration in the left infraorbital and nasal regions, associated with hypoesthesia and anesthesia of the ipsilateral gingival mucosa. These findings were clinically suggestive of vascular occlusion secondary to HA injection. Due to geographical constraints, the initial management was initiated remotely, including oral acetylsalicylic acid (200 mg), topical 2% nitroglycerin, mupirocin ointment, vigorous massage, and local heat application.

**Figure 1:**
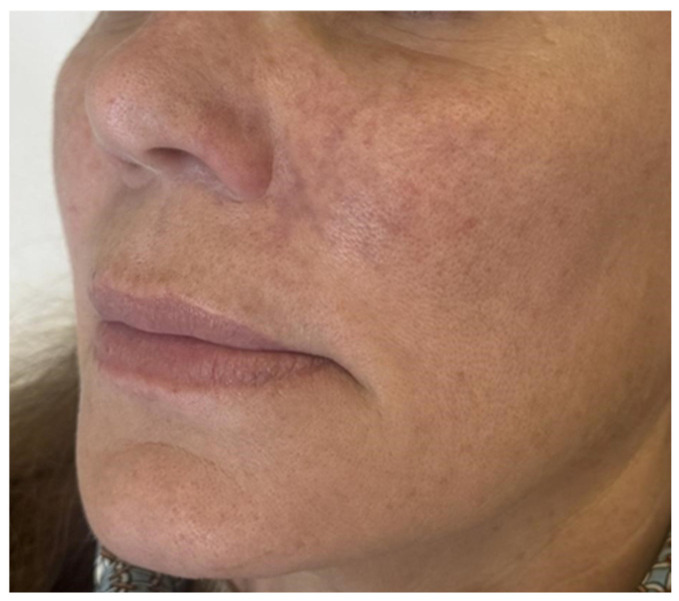
Livedo reticularis affecting the infraorbital and nasal regions.

**Figure 2:**
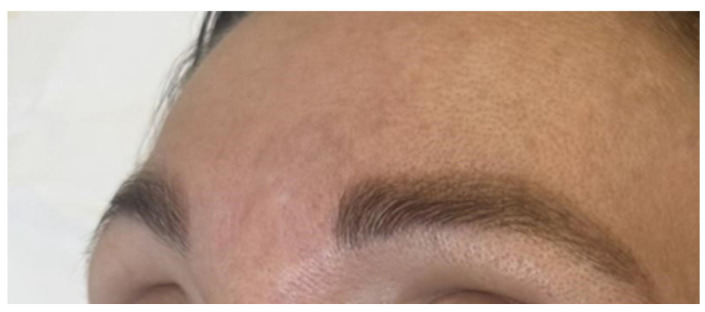
Livedo reticularis in the glabella region.

Twenty hours after the injection, the patient was re-evaluated in person. Clinical examination revealed livedo reticularis involving the left infraorbital region and nasal area, consistent with vascular compromise, as shown in Figure 1. Additional involvement of the glabellar region was observed, reflecting a wider distribution pattern within the affected vascular territory (Figure 2). Capillary refill was delayed (approximately 4 s) in the medial cheek, with associated tenderness on palpation. Persistent anesthesia of the left upper gingival mucosa was also noted, while capillary refill in the nasal and glabellar regions remained within normal limits (approximately 2 s).

Based on the distribution of clinical signs and the affected vascular territories, a diagnosis of infraorbital artery occlusion was established, with suspected predominantly anterograde embolization, although initial minor retrograde involvement of adjacent arterial branches was also considered. A low-dose hyaluronidase protocol was initiated. A solution of 1000 IU of hyaluronidase diluted in 10 mL of 0.9% saline (100 IU/mL) was prepared, and 200 IU (2 mL) were infiltrated along the infraorbital artery trajectory in a supraperiosteal plane. Immediate and significant improvement in capillary refill and tissue perfusion was observed following administration, which led to the decision to defer immediate ultrasound imaging.

**Figure 3:**
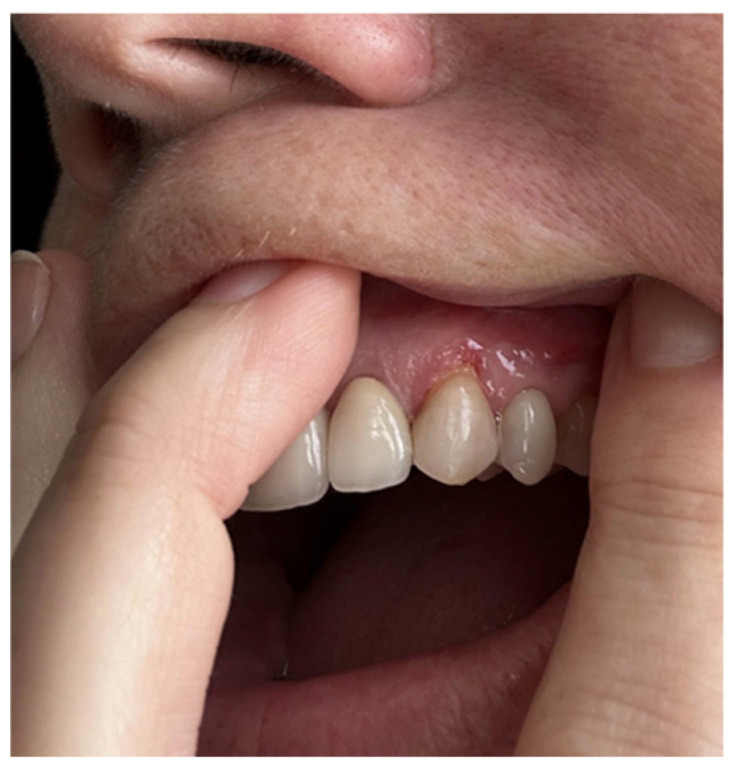
Ischaemic involvement of the gingival mucosa adjacent to the upper left canine.

**Figure 4:**
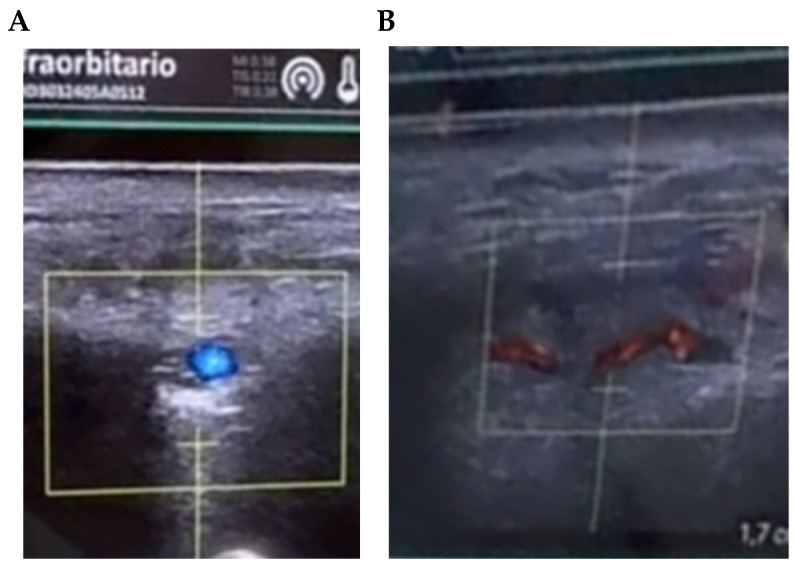
(**A**) Ultrasound image showing extrinsic compression of the infraorbital artery caused by hyaluronic acid deposits. (**B**) Ultrasound image demonstrating occlusion of the anterior superior alveolar artery consistent with retrograde embolization.

Twenty-four hours later, the patient developed a localized ischemic lesion in the gingival mucosa adjacent to the left upper canine, with persistent anesthesia, as illustrated in Figure 3. Given the atypical progression and mucosal involvement, a high-frequency ultrasound examination was performed using a high-frequency linear probe (18 MHz), including grayscale and color Doppler evaluation, allowing detailed visualization of soft tissues and vascular structures. Ultrasound assessment demonstrated hypoechoic periarterial HA deposits surrounding the infraorbital artery (Figure 4A), associated with reduction in Doppler flow signal compatible with extrinsic vascular compression. Additionally, absent Doppler flow was identified in the anterior superior alveolar artery territory, supporting secondary vascular occlusion (Figure 4B). Vessel trajectory, flow signal, and relationship between filler deposits and adjacent vascular structures were assessed dynamically in real time.

Based on the ultrasound evaluation, an additional 100 IU of hyaluronidase was administered under image guidance, specifically targeting the affected alveolar branch. This ultrasound-guided approach enabled precise delivery of the enzyme while avoiding unnecessary increases in the total administered dose.

Subsequent follow-up demonstrated complete restoration of tissue perfusion, with resolution of both cutaneous and gingival ischemia. Follow-up ultrasound evaluation performed the following day demonstrated restoration of flow in both the infraorbital artery and the anterior superior alveolar artery, with resolution of the previously observed thrombotic obstruction. A subsequent clinical and ultrasound reassessment performed one month later confirmed persistent arterial flow preservation and complete resolution of the gingival ulcerative lesion. No functional impairment or aesthetic sequelae were observed.

## 3. Discussion

Vascular occlusion remains the most severe complication associated with hyaluronic acid (HA) filler injections due to its potential to cause tissue necrosis, visual impairment, and permanent functional sequelae [4,7]. Although relatively uncommon, its clinical relevance is increasing in parallel with the growing number of aesthetic procedures performed worldwide [2,3]. Early recognition and prompt intervention are therefore essential to prevent irreversible tissue damage [6,13].

The pathophysiology of HA-related vascular occlusion may involve either intravascular embolization or extrinsic vascular compression, although differentiating between both mechanisms can be challenging in clinical practice [6,13]. In the present case, ultrasound findings in the infraorbital region were considered more consistent with extrinsic compression caused by periarterial HA deposits surrounding the infraorbital artery. However, the delayed appearance of gingival ischemia, together with ultrasound evidence of anterior superior alveolar artery involvement, suggested secondary embolic propagation affecting distal vascular territories [7,12]. Therefore, this case was interpreted as a combined vascular compromise pattern involving both extrinsic compression and secondary retrograde embolization. This resulted in ischemic compromise not only of the cutaneous territory but also of the gingival mucosa, an uncommon manifestation that has been rarely reported in the literature [12]. The medial malar region represents a particularly challenging anatomical area due to the presence of the infraorbital neurovascular bundle and the variability of facial vascular anatomy. Although sharp needles are commonly used for supraperiosteal filler placement in this region, some authors advocate the use of blunt cannulas in high-risk facial areas as a potential strategy to reduce the likelihood of inadvertent intravascular injection. Regardless of the technique employed, thorough anatomical knowledge, slow injection, low extrusion pressure, and careful product placement remain essential to minimize vascular complications.

Clinical diagnosis of vascular occlusion is primarily based on early recognition of warning signs such as disproportionate pain, livedo reticularis, pallor, and delayed capillary refill [6,8]. However, accurately identifying the affected vessel and determining the full extent of vascular involvement based solely on clinical examination can be challenging, particularly in anatomically complex regions such as the midface [9]. In this context, high-frequency ultrasound represents a valuable adjunctive tool, enabling real-time visualization of filler deposits, vascular structures, and tissue perfusion [10,11].

In the present case, ultrasound played a pivotal role not only in confirming the diagnosis but also in clarifying the underlying mechanism and extent of vascular compromise. Specifically, it allowed differentiation between extrinsic vascular compression by HA deposits and secondary arterial occlusion due to retrograde embolization. This level of diagnostic precision is difficult to achieve through clinical assessment alone and has important implications for therapeutic decision-making [10,11].

Hyaluronidase remains the first-line treatment for HA-related vascular occlusion, as it facilitates degradation and diffusion of the injected material [6,8]. Traditionally, high-dose protocols have been recommended to ensure sufficient enzyme diffusion and intravascular reach [7]. Several published protocols recommend repeated administration of high cumulative doses of hyaluronidase in cases of suspected vascular occlusion in order to maximize tissue diffusion and intravascular reach. In contrast, the total dose used in the present case (300 IU) was substantially lower than that frequently described in traditional protocols.

The selection of a low-dose hyaluronidase protocol in this case is supported by emerging evidence challenging traditional high-dose flooding regimens. A recent systematic review and pilot meta-analysis by Boey et al. [14] reported higher rates of complete tissue resolution with low-dose protocols compared with conventional high-dose approaches (88.1% vs. 69.7%). Similarly, Urso et al. [11] demonstrated that ultrasound-guided administration of relatively small amounts of hyaluronidase (30–150 IU) can successfully restore blood flow and achieve complete tissue recovery when the site of vascular compromise is accurately identified.

These findings support the concept that the enzymatic dose should be adapted to the estimated volume and location of the obstructing hyaluronic acid deposit rather than administered indiscriminately. In focal vascular occlusions, where the amount of filler causing the obstruction is typically limited, targeted low-dose administration may provide sufficient enzymatic activity while avoiding unnecessary tissue exposure.

In addition to its clinical effectiveness, a low-dose approach may offer safety advantages. Previous studies have suggested that adverse effects associated with hyaluronidase, including local inflammatory reactions, tissue irritation, and hypersensitivity responses, may be dose-dependent [14,15]. Therefore, when combined with ultrasound guidance, the use of the minimum effective dose may represent a rational strategy to maximize therapeutic benefit while minimizing treatment-related risks.

Based on this evidence, we propose a standardized approach for managing ischemic events across different facial zones: once the suspected artery is identified, an initial targeted dose of 200 IU (using a 100 IU/mL dilution) should be infiltrated along its trajectory, followed by immediate clinical reassessment. While this protocol is highly reproducible in other facial territories, in some cases, higher doses may be necessary if a larger vascular network or multiple adjacent arterial branches are involved. Furthermore, preventing such complications in the medial cheek requires strict adherence to safety guidelines. Our clinical practice for supraperiosteal hyaluronic acid injections includes delivering small boluses of a maximum of 0.2 mL per point using needles with a maximum thickness of 30 G (30 G or smaller), ensuring the needle remains strictly within the supraperiosteal plane without moving, thereby avoiding retrograde linear threads.

The use of ultrasound guidance enabled targeted enzyme delivery directly to the affected vascular territories, potentially reducing unnecessary exposure to high enzyme doses while maintaining therapeutic efficacy [7,11]. However, these approaches are associated with potential adverse effects, including excessive degradation of native HA, loss of tissue volume, and alterations in skin quality, described as post-hyaluronidase syndrome [16].

The integration of ultrasound into the diagnostic and therapeutic workflow offers several advantages. It allows early identification of vascular compromise, improves understanding of the mechanism of occlusion, and facilitates image-guided interventions [10,11]. Consequently, ultrasound-guided management may enhance treatment accuracy, reduce variability in clinical decision-making, and minimize the risk of overtreatment and associated complications.

Nevertheless, this report has several limitations. As a single-case observation, its findings cannot be generalized without caution. Additionally, the absence of standardized ultrasound parameters and quantitative perfusion assessment limits the ability to objectively compare outcomes. Further studies are required to validate the role of ultrasound-guided low-dose hyaluronidase protocols and to establish standardized diagnostic and therapeutic algorithms.

## 4. Conclusions

Infraorbital artery occlusion following hyaluronic acid filler injection may present with complex vascular involvement, including retrograde extension to the anterior superior alveolar artery and associated gingival ischemia. This case highlights the limitations of clinical assessment alone in accurately defining the extent and mechanism of vascular compromise. High-frequency ultrasound proved essential for precise diagnosis and enabled targeted, image-guided administration of low-dose hyaluronidase. Ultrasound-guided management may improve therapeutic accuracy, optimize treatment outcomes, and reduce the risk of unnecessary enzyme exposure and related adverse effects.

## Data Availability

Data supporting the findings of this study are available from the corresponding author upon reasonable request. Data are not publicly available due to privacy and ethical restrictions.
